# Mechanical Properties of Sugar Beet Roots under Impact Loading Conditions

**DOI:** 10.3390/ma16031281

**Published:** 2023-02-02

**Authors:** Paweł Kołodziej, Zbigniew Stropek, Krzysztof Gołacki

**Affiliations:** Department of Mechanical Engineering and Automation, Faculty of Production Engineering, University of Life Sciences in Lublin, 20-612 Lublin, Poland

**Keywords:** sugar beet root, mechanical impact, impact energy, contact volume, pendulum

## Abstract

Root damages due to mechanical impacts result in deterioration in commercial sugar beet quality. In order to determine the mechanical characteristics of roots, a stand equipped with a pendulum enabling impact investigations of whole beets was used. The roots were stored in a monitored environment for up to 5 days (temperature 15 ± 2 °C, 40 ± 2%). During the tests, the beets were struck against a flat steel resistant surface with the velocities *V_imp_* = 0.5, 1.0 and 1.5 m·s^−1^. The measurements of local root curvatures in three chosen impact areas and the deformation (*d_max_*) allowed modelling of the volume of contact (*CV*) by means of the ellipsoid cap. These investigations enabled the determination of the relations between the maximal impact force, *F_max_*, the impact energy, *E_imp_*, and the absorbed energy, *E_abs_*, as well as the contact volume and impact velocity, taking into account the root storage time, *S_t_*. It was found that the maximal impact force increased with increasing impact velocity and decreased with the storage time for each group of roots. With increasing velocity, there were also increases in the following: impact energy, absorbed energy, contact volume and maximal deformation, as well as absorbed energy, referred to as the mass *E_abs-v_* from *V_imp_*. The mean values of the stresses (*σ_max_*), being the quotients of the impact force (*F_max_*) and the surface area of the ellipsoid cap base (*A_BE_*), were 0.81–1.17 MPa, 1.064–1.59 MPa and 1.45–1.77 MPa for the velocities of 0.5, 1.0 and 1.5 m·s^−1^, respectively. It was confirmed that the statistical significance of the mentioned parameters changes depending on the impact velocity.

## 1. Introduction

The mechanization of production processes facilitates agrotechnical works during and after harvesting, including sorting, packing or transporting processes, which can result in various kinds of biological material defects [1,2,3]. This takes place because in many cases, mechanical damages are a result of impact character loads. Permanent, most often local, interior and exterior damages cause decreases in the following values:

- Consumption of, e.g., apples, peaches, nectarines, plums, etc., as well as tomatoes or potatoes [4,5,6,7].
- Industrial (e.g., sugar beets or potatoes) [7,8,9].

Visible damages on plant products (e.g., fruits and vegetables), most frequently bruising, can already occur at a velocity of 0.25 m·s^−1^, and their size can increase with increasing motion velocity that is caused by, e.g., picking up, sorting, transport mechanisms or drop height during transshipment [10,11,12]. The identification of root defects in the forms of cracks, breakages (fractures) and abraded surfaces during a beet’s movement inside a harvester, e.g., on transporters and cleaning turbines, is discussed in [13,14,15], along with the necessity to avoid these defects. Methods allowing the obtaining of information about biological material responses to mechanical loads, particularly impact loads, have been searched for. The literature reports the descriptions of various impact tests, which are divided into a small number of types. Experiments using a physical pendulum can be conducted on samples [16] or on whole fruits. Experiments that involve striking a fruit placed at the end of an arm against a flat surface [17,18,19,20,21] or striking an immobile fruit with an impactor fixed to the pendulum [22,23,24] are more frequently applied. Another type of test involves free dropping from a chosen height; this is used for estimations of the fruit’s characteristics, e.g., guava ripeness [25] or apples’ resistance to damages [26]. Such investigations have determined the results of loads during the impact of various materials against surface areas [27] and the elasticity modulus and maximal forces using a triaxial acceleration sensor for potato tubers [28]. 

As was already mentioned, impact tests enable the estimation of mechanical resistance affecting the extent of damages and, as a result, consumption attractiveness where the appearance of the fruit or vegetable is an essential factor. The preliminary identification of damages to a fruit’s cellular structure is made more frequently, by visually inspecting for exterior or interior blue spots after bruising. Damage extent measurements can then be made in relation to three mutually perpendicular axes, which allows for modelling of the blue spots’ volume [29,30]. These actions are undertaken during estimates of visible damages not only to fruits (apples, pears, peaches, avocados, etc.), but also to potato tubers. However, for the hidden (interior) blue spots, time-consuming and destructive biochemical or physical methods are applied [31].

In the case of sugar beets, the values of parameters such as the Young modulus, the Poisson ratio, failure stress and failure strain are of significant importance in determining storage and processing applicability. Due to the possibility of monitored loading in time as well as the accepted reproducibility of research results, quasistatic tests on samples are usually carried out [32,33,34]. In a paper by Nedomova et al. [35], samples cut from the beets were subjected to compression and puncture. The authors found that root failure strength increases with storage time, and it is more advantageous to take the results of a compression test rather than a puncture test to describe mechanical parameters. The puncture, compression and bending tests allowed differentiation of sugar beet varieties with respect to tissue strength. However, the bending test showed great variation between repetitions. Therefore, it is not recommended for the characterization of sugar beet varieties [36,37]. 

Destructive impact studies carried out with the use of a heavy pendulum on samples confirmed the importance of the criterion of critical stresses for sugar beet roots [38]. The values of the energy needed for damaging the sample as well as that absorbed by it during the impact decreased with storage time. The value of critical strain was three times smaller than the value reported in the literature, but it was obtained under quasistatic load.

Another method of determining the mechanical properties of roots is nondestructive investigation, e.g., using a laser vibrometer. Tests involving the use of stored roots are presented in a paper by Trnka et al. [39]. The authors determined the beet rigidity from the spectrum of point vibration frequency on the root-side surface after impact from a cylindrical rod. The changes in impact impulse depending on the storage time showed that the maximal force increases with the impact velocity and decreases with the storage time. Studies aiming at the prediction of various sugar beet properties by means of spectroscopy in the range of visible light and near-infrared are presented in a paper by Pan et al. [40]. The maximal destructive force for a given deformation, its surface area under the force–dislocation curve and its slope were determined. This method’s effectiveness for determining sugar and moisture content was confirmed. However, it was insufficient for the estimation of the mechanical parameters determined using the compression test. 

The investigation results and their interpretations of the mechanical properties of sugar beets presented in the above review indicate the essential “critical points” at which the roots can undergo various kinds of loads. Taking into account the velocity changes during the beet’s movement in the harvester’s interior sections (e.g., cleaning turbines), it is important to determine the root response as a whole to impact loads at different impact velocities. 

The objective of this paper is the determination of the mechanical characteristics of sugar beet roots under impact load conditions and their dependence on impact velocity. The research stand used for this investigation was composed of two independent parts, impact-forcing and measuring parts, which allowed the following to be obtained:

- Time courses of the beet root’s reaction and force response to the impact.
- The course of deformation during the impact in time as a sequence of images.

The obtained courses enabled the determination of the course of reaction in terms of force displacement, maximal impact force, impact energy, root-absorbed energy, beet deformation, maximum contact area and contact volume. It was possible to determine the derived quantities of such derivatives as maximal stresses in the contact area and energy absorbed for the root mass unit. The effect of storage time on the mechanical characteristics was determined by repeating the investigations over a period of five days after the harvest and measuring the moisture content in the roots. 

## 2. Materials and Methods

### 2.1. Materials 

The investigations were carried out using sugar beets of the Everest variety obtained from a local sugar factory, with root masses ranging from 817.6 to 1662.4 g. The beets’ mass was measured using the laboratory scales FAWAG PM-3/1 with a measurement range of 0–3000 g and a scale interval of 0.1 g. The roots used for the investigations were taken directly from fields in the Lublin region (Lublin, Poland) and were stored in closed containers for 1, 2, 3, 4 or 5 days at a temperature of 15 ± 2 °C and humidity of 40 ± 2%, with the surrounding parameters being controlled every day.

### 2.2. Research Stand

**Figure 1:**
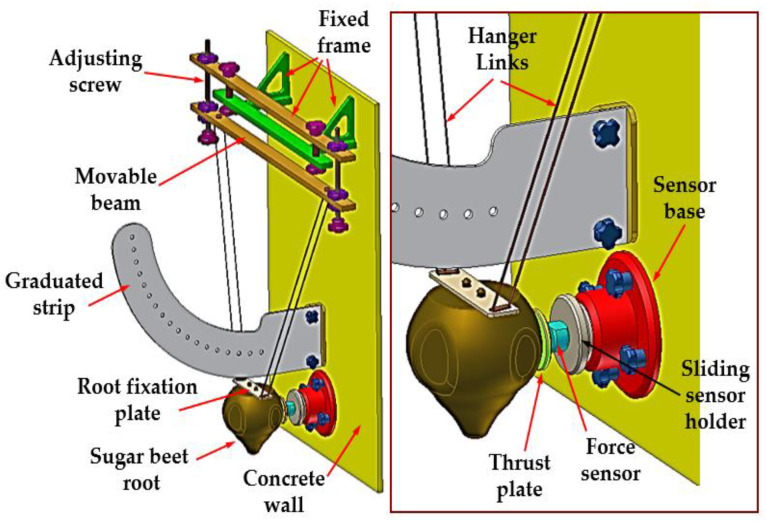
Scheme of the stand for the impact tests of sugar beet roots.

The tests employed a research stand (Figure 1) in the form of a pendulum built with a fixed frame attached to a wall, to which a movable beam with plastic hanger links and a root fixation plate were also attached. The beam and links formed a 1 m long triangular hanger. 

The roots were fixed to the plate with screws of large-pitch thread. Due to a much smaller mass of hanger links compared with the root mass, it can be stated that the beet mass is decisive for the value of inertia moment calculated in relation to the rotation axis of these elements. The force sensor was connected to the movable holder and placed in the concrete wall base. The connection was blocked by the screw grip. The shift of holder in the package ensured regulation of root position in relation to the sensor so that the force at the time of impact had a horizontal direction. The resistance plate was fixed to the force sensor. A suitable beet root setting was possible using the regulating screws in the movable beam connected with the pendulum arm. These allowed for the dislocation of the root fixed to the plate and for hanging it on the links in both perpendicular and horizontal directions, enabling precise setting up in the sensor axis.

### 2.3. Volume of Deformation of the Root

Modelling the shape of fruit impact bruising has been taken into consideration by many authors. The bruising volume (BV) in the form of a blue spot was described as one or a composition of two solids using the formulae for shape calculation, such as for a segment of sphere or ellipsoid [30]. Using this method, the apples’ [1,3,27], pears’ [29], peaches’ and nectarines’ [11,21], pomegranates’ [41] and coconuts’ [42] deformations were modeled. As already mentioned, there were no blue spots in the sugar beet after the impact. This made determination of the total damage volume difficult. Therefore, it was assumed that the spatial deformation confined by the root curvature and a vertical plane corresponding to the maximal horizontal deformation of the beet during the impact created the local bruising. The modelling of the beet’s contact with the resistant surface of the force sensor begins with determination of characteristic dimensions, which enable determination of closed curves assigned to the bruising volume boundaries.

**Figure 2:**
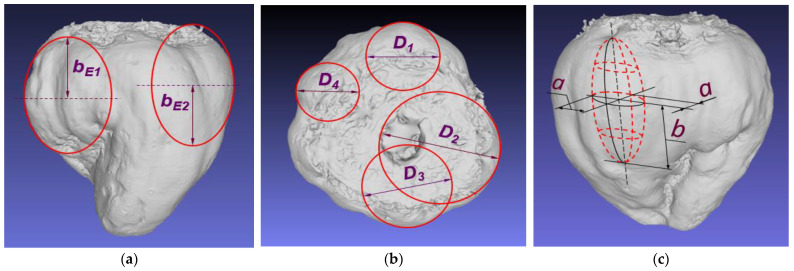
Three-dimensional scans of the sugar beet. (**a**) View in the plane parallel to the main axis with large half-axes (*b_E_*_1_, *b_E_*_2_) of the ellipses describing the vertical root curvatures; (**b**) view in the plane perpendicular to the main axis and diameters (*D*_1_*–D*_4_); (**c**) local beet surface curvatures modelled by the prolate ellipsoid of the dimensions; half-axes—small *a* and large *b*.

Figure 2a,b presents 3D scans of the exemplary roots and lines for description of the surface curvature in the vertical and horizontal planes. As the beet possesses a surface of small vertical curvatures (flat arcs), an attempt was made to describe it by means of ellipses of large *b_E_*_1_*, b_E_*_2_ half-axes (Figure 2a).

However, local horizontal curvatures can be determined by means of circles of the diameters *D*_1_*–D*_4_ (Figure 2b) using a radius measuring device. Such interpretation allows reconstruction of local root geometry as a prolate ellipsoid on the half-axes *a* and *b* (Figure 2c), whose characteristic features are circular cross-sections in the plane perpendicular to the large half-axis *b*.

**Figure 3:**
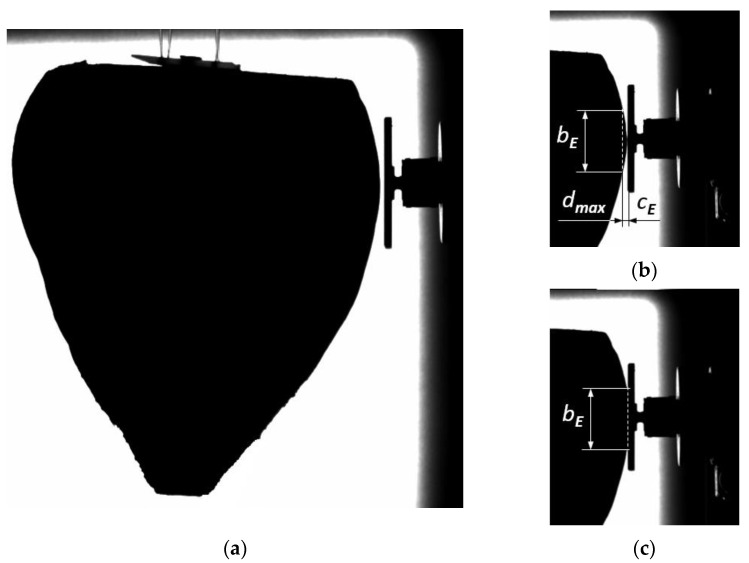
Image sequences of the root using the camera Phantom Miro M320 during the impact test. (**a**) The beet and resistance plate fixed to the force sensor; (**b**) the impact start; (**c**) the impact end; *d_max_*—the pictorial determination of maximal deformation *d_max_ = c_E_*.

The diameter of the largest ellipsoid circle can be determined as the double value of the small half-axis 2*a = D*_1_. During impact, the root curvature is in contact with the flat resistant surface of the sensor (Figure 3a). The value of the formed deformation, *d_max_*, can be determined in millimeters during the test (Figure 3b,c). Figure 3c presents the impact in the final phase in which the deformation reaches the maximal value *d_max_*. The value *b_E_* in the picture corresponds to this deformation. In order to show the maximal deformation *d_max_ = c_E_*, the deformation was moved to Figure 3b, which presents the beginning of the root surface contact with the sensor-resistant plane.

**Figure 4:**
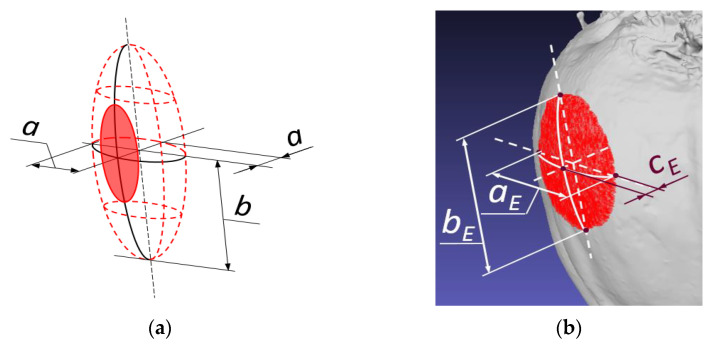
Modelling the contact volume (*CV*) as the ellipsoidal cap. (**a**) The ellipsoid dimensions; (**b**) the ellipsoid cross-section dimensions: *a_E_*, *b_E_*, *d_max_ = c_E_*.

The values of dimensions describing the deformation volume—*a_E_, b_E_* and *d_max_ = c_E_* (in millimeters)—are much smaller than the half-axes *a* and *b* of the prolate ellipsoid used for reproduction of the local root shape (Figure 4a). In this paper, there was an attempt to model contact volume (*CV*), total deformation during the impact, as the ellipsoid cap (Figure 4), which can be expressed using the following dependence:

(1) $$CV=\frac{\pi \cdot b}{3a}\cdot c_{E}^{2}\cdot \left(3a-c_{E}\right)\;$$

where *c_E_ = d_max_*—the horizontal deformation, mm; *a*—the small ellipsoid half-axis; 2*a = D*_1_ mm, *b*—the large ellipsoid half-axis, mm.

**Figure 5:**
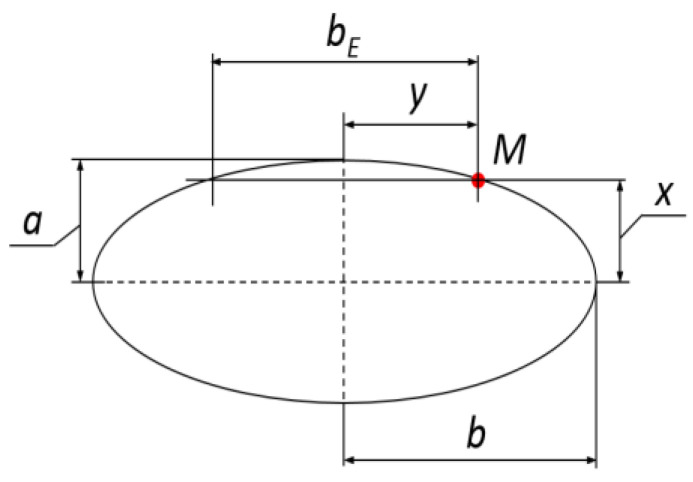
The largest ellipsoid cross-section with auxiliary denotations for determination of the large half-axis *b* value.

For determination of the volume *CV*, one requires real values of the parameters *a* and *d_max_ = c_E_* which can be obtained from direct measurements, e.g., 2*a = D*_1_, and based on the results of impact tests as the maximal deformation *d_max_ = c_E_* (Figure 3b). However, the value of the large half-axis *b* of the prolate ellipsoid presented in Figure 4a can be calculated from the dependence determining the position of point *M* fulfilling the ellipse equation. Figure 5 presents the ellipse, which is also the largest longitudinal cross-section. The ellipse equation is as follows:

(2) $$\frac{x^{2}}{a^{2}}+\frac{y^{2}}{b^{2}}=1$$

where *x = a—c_E_*, *y* = **½** *b_E_*—the coordinates of the ellipse *M* point position. 

The coordinates of the point *M* on the ellipse (cross-section profile), *x = a − c_E_* and *y* = ½·*b_E_*, were used for determination of the value of large half-axis *b* from the following equation:

(3) $$b=\sqrt{\frac{y^{2}}{1-\frac{x^{2}}{a^{2}}}}=\sqrt{\frac{{\left(\frac{1}{2}b_{E}\right)}^{2}}{1-\frac{{\left(a-c_{E}\right)}^{2}}{a^{2}}}}=\sqrt{\frac{a^{2}\cdot b_{e}^{2}}{4\cdot \left(a^{2}-{\left(a-c_{e}\right)}^{2}\right)}}\;$$

where *a*—the small half-axis of the ellipse; *c_E_*—the maximal deformation.

The measured root curvatures and the surface deformations registered during the tests allowed calculation of the assumed contact volume. The next step was determination of *CV* from dependence (1) and taking this as the area impact effect. The calculated value *CV* did not take into account the effects of impact out of the plane defined by the dimension of deformation *c_E_*, because the exact volume of their occurrence is not known due to the lack of visible blue spots on the root tissue. Cross-section area of ellipsoid based on ellipse (*A_BE_*) with the main axes *a_E_* and *b_E_*, presented in Figure 4b, was obtained from the following equation:

(4) $$A_{BE}=\frac{\pi }{4}b_{E}\cdot a_{E}$$

where *a_E_*—the width of the ellipsoid section; *b_E_*—the height of the ellipsoid section. 

Taking into account the maximal values of the root reaction force (*F_max_*) for the mechanical impact obtained from the tests as well as *A_BE_* determined from the relation (4), normal compressive stresses of a dynamic character formed during the root impact against the plate surface were calculated:

(5) $$\sigma_{max}=\frac{F_{max}}{A_{BE}}$$

where *σ_max_*—the maximal stress, MPa; *F_max_*—the maximal value of impact force obtained from the tests, N; *A_BE_*—the area of ellipsoid based on ellipse, mm^2^.

### 2.4. Moisture Content

After the impact tests, 6 cylindrical samples of a diameter of 11 mm and a length of 14.5 mm were cut out from each root from the impact place’s surroundings. The samples were then weighed using the laboratory scales AS 110.R2 of a measurement range of 110 g and an elementary interval of 0.0001 g, and then dried according to the procedure ASAE [43]. A chamber for thermal studies, KBC-65W (WAMED, Poland), was used for drying. After the required time, the dried samples were weighed again and the root moisture defined as the liquid content in the wet material was calculated from the following equation:

(6) $$WSB\%=\frac{M_{k}-M_{b}}{M_{b}}\cdot 100$$

where *WSB%*—the moisture content; *M_k_*—the mass of root sample in grams before drying, g; *M_b_*—the mass of the sample after drying, g.

### 2.5. Apparatus and Slotted Section

The measurements of the force of root impact against a metal plate were made using a No 2311-10 sensor—produced and calibrated by the firm Endevco (Depew, New York, NY, USA), with a voltage sensitivity of 10 mV·lbf^−1^ (0.44 mV·N^−1^—typical voltage sensitivity) and measurement range of 2200 N—which is a piezoelectric transducer that generates an analog signal proportional to the force being measured. The signal from the force sensor was transmitted using the recorder LMS SCADAS Mobile (Siemens, Munich, Germany) to a computer equipped with LMS Test.Xpress 8A software for registration and analysis of the data, which could then be visualized, processed and recorded to a hard disk in real time. The measurement initiation took place when the value of root reaction force exceeded 0.5 N at the frequency of 10.24 kHz. A Phantom Miro M320 camera (Vision Research, Wayne, NJ, USA) with a lens with 25 mm focal length was used for recording the course of root impact against the plate surface perpendicular to the direction of pendulum movement. The sequence of images was analyzed using Phantom Camera Control (PCC-2) software with pixel resolving power of 1024 × 768 at the velocity of 3413 frames per second. Figure 3 presents time-lapse photos obtained by means of the camera showing the beet root before and during the impact against the flat immovable surface of the plate fixed to the force sensor. The course of root velocity and its deformation in time were determined using Tema Motion Vision 3.8 software (Image Systems, Linköping, Sweden).

### 2.6. Measurements

**Figure 6:**
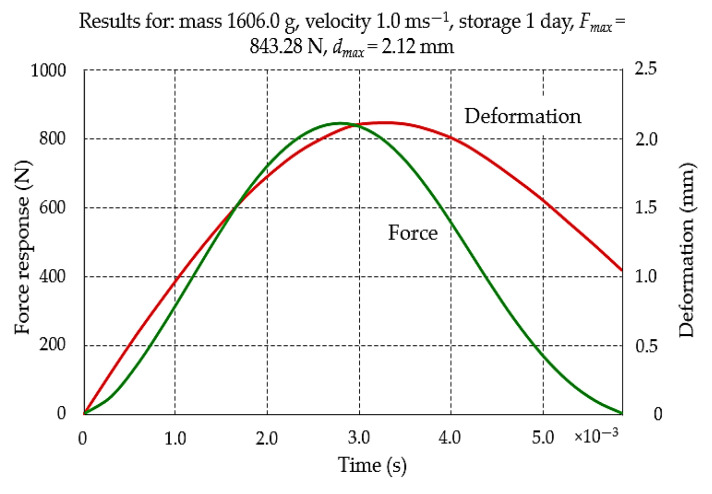
Typical force response in time and deformation in time curves during the impact of sugar beet root against the rigid plate.

During the tests, beets were dropped from heights of about 13 mm, 80 mm and 115 mm, obtaining impact velocities of 0.5, 1.0 and 1.5 ms^−1^, respectively. In a single research cycle involving 5 beets, the values of reaction and deformation forces in the root surface were obtained, and their exemplary courses in time are presented in Figure 6.

**Figure 7:**
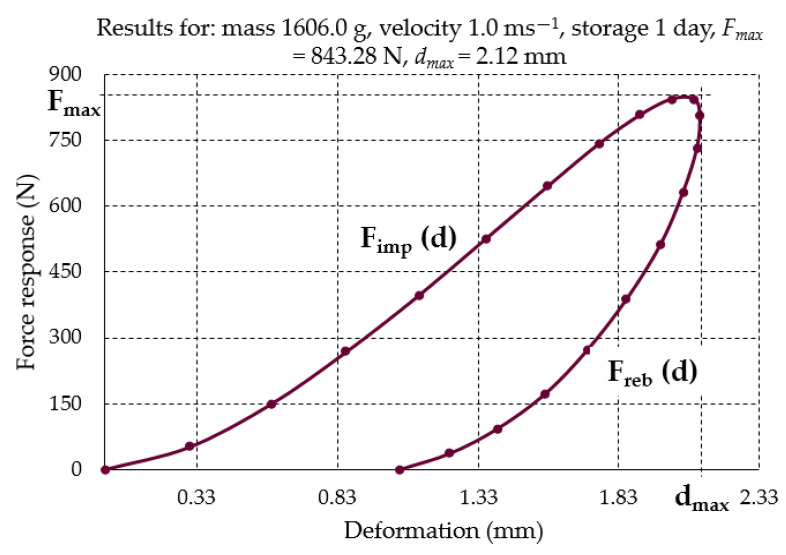
Typical relationship between the force response and the deformation during the impact and the rebound of sugar beet root.

Knowing the courses of response and deformation forces in the function of time, it was possible to determine the dependences of reaction forces in the function of root deformation (Figure 7). These allowed for calculating the impact (*E_imp_*) and elastic rebound (*E_el_*) energies as well as absorbed energy (*E_abs_*) by the root using the following formulae:

(7) $$E_{imp}=\overset{d_{max}}{\underset{0}{\int }}F_{imp}\left(d\right)\;d\left(d\right)$$

(8) $$E_{el}=\overset{d_{max}}{\underset{0}{\int }}F_{reb}\left(d\right)\;d\left(d\right)$$

(9) $$E_{abs}=E_{imp}-E_{el}$$

where

*E_imp_*—The energy of the mechanical impact J,
*E_el_*—The energy of beet material response to the mechanical impact J,
*E_abs_*—The energy absorbed during the mechanical impact (the surface area between the curves *F_imp(d)_* and *F_reb(d)_* in the part from *d =* 0 to *d = d_max_*).

The absorbed energy can be mainly recognized as the energy of stable deformation causing destruction of cellular (tissue) structures during mechanical impact. Its value can depend on experimental parameters such as root mass, impact velocity and storage time. To obtain the quantity which makes the value of absorbed energy independent of the root mass, the indicator *E_abs-v_*, which is the quotient of *E_abs_* and the root mass m, was proposed:

(10) $$E_{ab-v}=\frac{E_{abs}}{m}$$

where *E_ab-v_*—the absorbed energy related to the mass, J·g^−1^; *m*—the root mass, g. 

## 3. Results and Discussion

**Figure 8:**
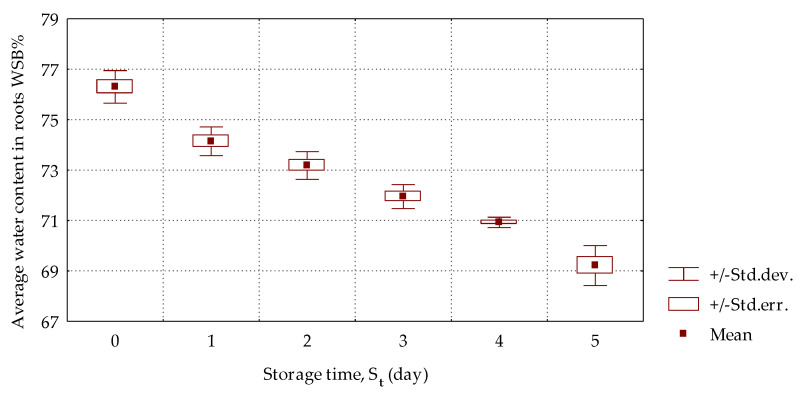
Average water content, *WSB%*, in the beet roots in the surrounding impact sites depending on the storage day.

The water content (*WSB%*) in six samples cut out from the roots in the surrounding impact sites, which was determined based on Equation (5), was averaged and is presented in relation to the storage day in Figure 8. 

A decrease in water content in the studied samples in the analyzed storage period was observed. Moreover, a statistical dependence between the moisture content and storage time with a correlation coefficient of *R*^2^ = 0.94 was found.

**Figure 9:**
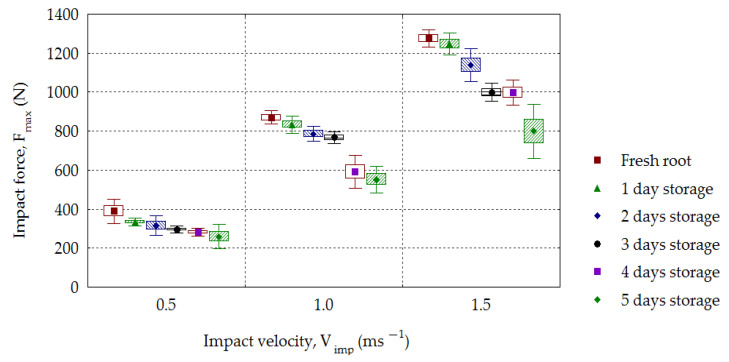
Diagram of maximal impact force (*F_max_*) dependence on impact velocity (*V_imp_*) for the stored beets.

In the impact tests, the height of the drop increases the impact velocity; therefore, the obtained dependence of the maximal impact force on velocity can be directly attributed to the drop height. Figure 9 presents the dependence of the maximal force of root response *F_max_* on the impact velocity *V_imp_*, taking the storage time into account. An increase in maximal values of forces with increasing impact velocity was found for all groups of roots stored from 0 to 5 days. The maximal impact force reached the following mean values for fresh roots: 389.3 N for 0.5 m·s^−1^ velocity, 870.5 N for 1.0 m·s^−1^ and 1274.4 N for 1.5 m·s^−1^. The values of forces decreased on successive days for all impact velocities, and *F_max_* reached the following mean values for 5-day-old roots: 259.0 N for the velocity 0.5 m·s^−1^, 552.2 N for 1.0 m·s^−1^ and 799.5 N for 1.5 m·s^−1^. The dependence of the maximal value of force, *F_max_*, on the velocity, *V_imp_*, and the day of storage, *S_t_*, was statistically significant and the determination coefficients were *R*^2^ = 0.87–0.98 and *R*^2^ = 0.49–0.79, respectively. The increase in the value of the maximal impact force with increasing impact velocity was confirmed by impact studies carried out not only for beets [24,33], but also for potatoes [44], peaches [45], corn [46], blueberries [47], apples [27,48,49,50], pears [29], kiwifruit [51] and guava [25]. This results from the viscoelastic nature of plant materials that are vegetables and fruits, and is connected with the loss of turgor in the tissues. 

Turgor is defined as a state of cellular wall tension caused by the hydrostatic pressure inside the cell. Its effects are, e.g., compactness and rigidity as well as the possibility of shape conservation. Assuming that while beets are being ploughed, the cell vacuole is filled with water at the highest pressure—in other words, they possess the maximal turgor—then at each later state (after the storage) they will lose water due to its flow outside the cell and then outside the root, e.g., during transpiration. As the beet “softens” from the outside, the drops of moisture in the roots proceed faster in the cork, pericycle and phelloderm cells as well as in the cambium and conducting bundles, which are the exterior coating in the root. Depending on the root diameter, the total thickness of these layers can differ, but for the most common values, which are in the range of 120–130 mm and to the depth of 20 mm, the surface structure is as described above. Thus, it can be stated that in the impact tests using whole roots, the layers of cork and endoderm as well as phelloderm and cambium transfer the impact loads. Therefore, the moisture reduction in the exterior layers affects the root response force values during mechanical impact. The mean content of moisture, *WSB%*, in the exterior root layers changed with the storage time, and under the conditions of the 5-day experiment it decreased from 76.3% to 69.2% (Figure 9).

**Figure 10:**
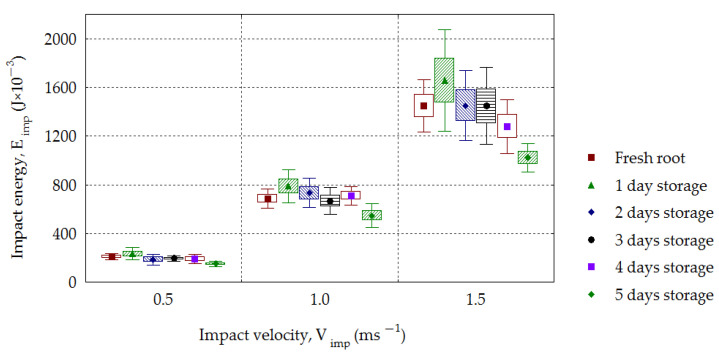
Dependence of impact energy (*E_imp_*) on the impact velocity (*V_imp_*) for the stored beets.

**Figure 11:**
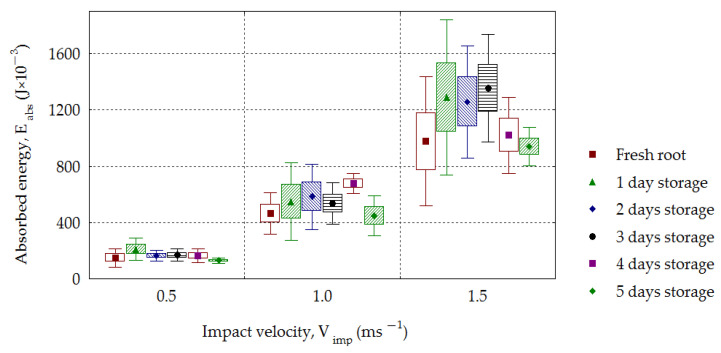
Dependence of absorbed energy (*E_abs_*) on the impact velocity (*V_imp_*) for the stored beets.

**Figure 12:**
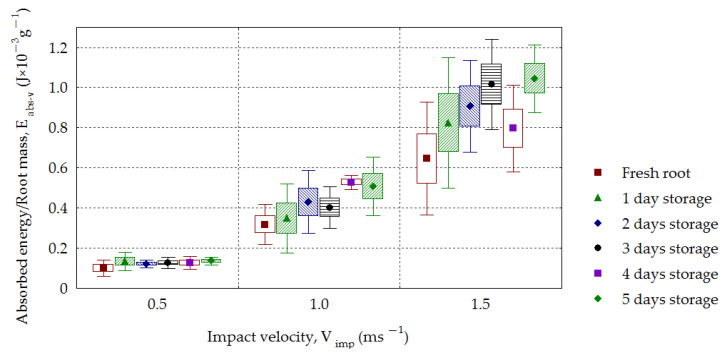
Dependence of absorbed energy related to mass (*E_imp-v_*) on the impact velocity (*V_imp_*) for the stored beets.

The increase in impact energy with the increasing drop height for potatoes is discussed in papers by Azam and Eissa [52] as well as by Stropek and Gołacki [44]. This also applies to sugar beets, for which the values of impact energy *E_imp_* increased with the increasing impact velocity *V_imp_* for all groups of stored roots in the period of time from 0 to 5 days (Figure 10). The impact energies *E_imp_*, e.g., for fresh roots, had the values 0.188–0.249 J, 0.578–0.801 J and 1.223–1.778 J, respectively, depending on whether the drop velocity *V_imp_* was 0.5, 1.0 or 1.5 m·s^−1^, and were statistically significant, whereby *R*^2^ = 0.85–0.95. For the dependence shown in Figure 11 concerning the increase in the absorbed energy *E_abs_* with the increasing impact velocity *V_imp_*, statistical significance was found for all groups of stored roots (*R*^2^ = 0.63–0.95). The similar relationship is observed in case of *E_abs-v_* quantity (Figure 12). The presented research results can be compared with the effects of experiments on impacts involving pomegranate, based on which Shafie et al. [41] found that the energy absorbed during the impact had the greatest effect on the bruising volume.

**Figure 13:**
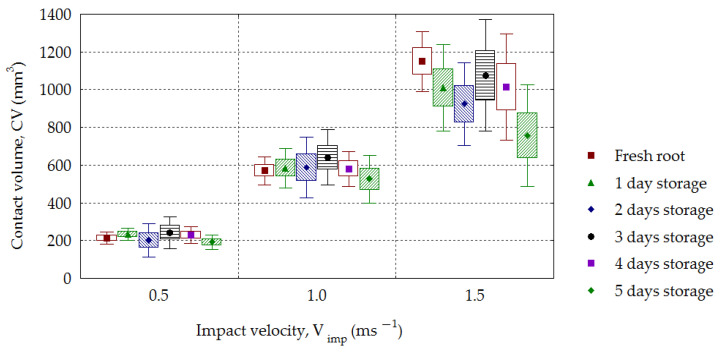
Dependence of the contact volume (*CV*) on the impact velocity (*V_imp_*) for the stored beets.

**Figure 14:**
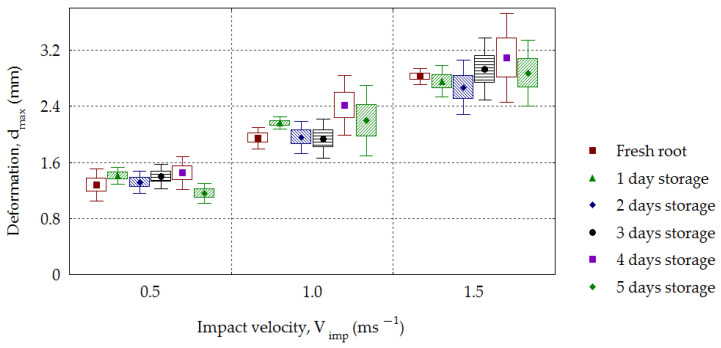
Dependence of the maximal deformation (*d_max_*) on the impact velocity (*V_imp_*), taking the storage time (*S_t_*) into account.

The dependence of the contact volume *CV* on impact velocity *V_imp_* for sugar beets is presented in Figure 13. A *CV* increase with increasing impact velocity was observed for all analyzed groups of roots, and this dependence was statistically significant (*R*^2^ = 0.68–0.93). From the dependence of the absorbed energy referred to as the mass *E_abs-v_* on the impact velocity *V_imp_* calculated from Formula (9), an increase in values (0.110–0.137 J·g^−1^, 0.317–0.527 J·g^−1^ and 0.645–1.044 J·g^−1^) was obtained for successive impact velocities. Statistical significance for the analyzed dependence *E_abs-v_* on *V_imp_* (*R*^2^ = 0.66–0.90) and for *V_imp_* = 1.0–1.5 m·s^−1^ was also found on the storage day *S_t_*. From analyzing the effect of impact velocity *V_imp_* on the amount of maximal deformation, *d_max_*, the results presented in Figure 14 were obtained. With the increasing impact velocity, the deformation increased for all groups of stored beets. The statistical significance of the effect of the impact velocity *V_imp_* on the amount of maximal deformation, *d_max_* (*R*^2^ = 0.74–0.92), was observed. This tendency presented in beets has been confirmed by other investigations. Ahmadi et al. [53] analyzed the behavior of various layers (skin, flesh and seed chamber) during impacts of an apple against another apple and apples against a rigid object using the finite elements method. The maximal deformation was larger at higher impact velocities in each tested layer of apples. Lu and Wang [54] determined the limit for bruising of “Gala” apples to be in the drop height range of 0.04–0.7 m. The maximal deformation increased with increasing drop height. The characteristic feature of these studies was finding the zero values of deformation at the drop height of 0.04 m, which was interpreted as the critical height at which damage takes place. The increase in maximal deformation with increasing impact velocity was found for three varieties of apples and two varieties of pears [19,55].

**Figure 15:**
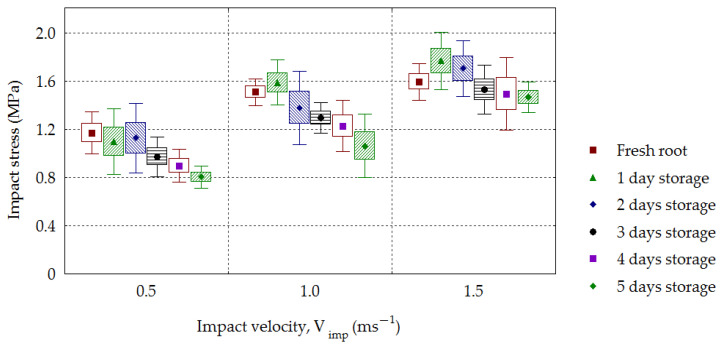
Dependence of the maximal impact stress (*σ_imp_*) on the impact velocity (*V_imp_*), taking the storage time (*S_t_*) into account.

Figure 15 presents a statistically significant increase in the maximal stress *σ_max_* with increasing impact velocity *V_imp_* (*R*^2^ = 0.47–0.69). In the case of fresh roots, a slight increase in stress was observed between velocities of 1.0 and 1.5 m·s^−1^. This is consistent with the results of studies on pears and potatoes, for which the stress increase tends to stabilize after exceeding a certain impact velocity [19,45]. The calculated mean values, *σ_max_*, decreased with the storage time, *S_t_*, and were in the ranges of 1.170–0.804 MPa, 1.589–1.063 MPa and 1.769–1.450 MPa for velocities of 0.5, 1.0 and 1.5 m·s^−1^, respectively. This dependence was statistically significant and the determined general tendency towards a decrease in maximal stress with increasing storage time is confirmed in a paper by Vursavus and Ozguven [56]. 

It was not possible to determine the extent of interior root damages of a permanent plastic character based on the obtained results. However, an increase by the magnitude of several times in the value of the absorbed energy indicator for the mass unit *E_abs-v_* with increasing velocity indicates the possibility of exceeding the elasticity limit in some areas of the root and the appearance of plastic deformations. Similar conclusions can be drawn by observing the tendency towards stabilization of the impact stress values with the increasing impact velocity. In this case, this can evidence the approximation of the mean stress values to the critical value, which should be stable and independent of the impact velocity (dynamic yield pressure). The precise determination of critical stress values under impact load conditions, including stress propagation in the wave form, requires further detailed investigations.

## 4. Conclusions

Based on the impact tests of the whole roots using a high-speed camera and a light link pendulum, the courses of beet force response in time were obtained and the sequences of root impact against a flat resistant surface were registered. 

The maximal impact force achieved the following mean values for the fresh roots: 389.3 N for the velocity 0.5 m·s^−1^, 870.5 N for 1.0 m·s^−1^ and 1274.4 N for 1.5 m·s^−1^. In the successive days of storage, *F_max_* decreased, achieving the following mean values for the 5-day-old roots: 259.0 N for the velocity 0.5 m·s^−1^, 552.2 N for 1.0 m·s^−1^ and 799.5 N for 1.5 m·s^−1^. The increase in the maximal value of the root force response *F_max_* with the increasing impact velocity *V_imp_* is evidence of the viscoelastic properties of the studied material.

The impact energy *E_imp_* increased with increasing impact velocity and decreased for each storage day. The mean values in the period of 0–5 days were in the following range: 233.6–149.0 (10^−3^ J) for the velocity 0.5 m·s^−1^, 787.7–547.2 (10^−3^ J) for 1.0 m·s^−1^ and 1656.6–1022.1(10^−3^ J) for 1.5 m·s^−1^. The value of absorbed energy increased depending on the impact velocity. The energy, *E_abs_*, reached a mean of 208.6–128.9 (10^−3^ J) for the velocity of 0.5 m·s^−1^, 678.5–447.8 (10^−3^ J) for 1.0 m·s^−1^ and 1288.2–938.1 (10^−3^ J) for 1.5 m·s^−1^. With increasing impact velocity, an increase in the root maximal deformation was observed, with the following values: 1.16–1.45 mm for *V_imp_* = 0.5 m·s^−1^, 1.94–2.41 mm for 1.0 m·s^−1^ and 2.67–3.09 mm for *V_imp_* = 1.5 m·s^−1^. A similar dependence was found in the case of contact volume *CV*. An increase in *CV* was observed with the increasing impact velocity for all analyzed groups of roots, and this dependence was statistically significant (*R*^2^ = 0.68–0.93). 

Studying the dependence of the absorbed energy referred to by the mass of *E_abs-v_* on the impact velocity *V_imp_* calculated from Formula (9), increased values were obtained in the ranges of 0.110–0.137 J·g^−1^, 0.317–0.527 J·g^−1^ and 0.645–1.044 J·g^−1^ for successive impact velocities (*R*^2^ = 0.66–0.90). It was found that the values of maximal stresses, *σ_max_*, increased with the increasing impact velocity and decreased depending on the storage day. The calculated mean values of *σ_max_* decreased with the storage time, being 1.170–0.804 MPa, 1.589–1.063 MPa and 1.769–1.450 MPa for the velocities 0.5, 1.0 and 1.5 m·s^−1^, respectively. 

The average moisture content, *WSB%*, in the exterior root layer of the root changed with the storage time and decreased from 76.3% to 69.2% under the conditions of the 5-day experiment. Therefore, it can be stated that the decrease in the maximal values of the beet response forces (*F_max_*) against the impact as well as the changes in the impact energy (*E_imp_*), absorbed energy (*E_abs_*) or maximal stress (*σ_max_*) are directly connected with the loss of water in the root surface part. 

## Data Availability

Not applicable.
